# Unraveling the causes of adaptive benefits of synonymous mutations in TEM-1 β-lactamase

**DOI:** 10.1038/s41437-018-0104-z

**Published:** 2018-07-02

**Authors:** Mark P. Zwart, Martijn F. Schenk, Sungmin Hwang, Bertha Koopmanschap, Niek de Lange, Lion van de Pol, Tran Thi Thuy Nga, Ivan G. Szendro, Joachim Krug, J. Arjan G. M. de Visser

**Affiliations:** 1 Institute for Theoretical Physics, University of Cologne, Cologne, Germany; 2 Laboratory of Genetics, Wageningen University, Wageningen, The Netherlands; 3 Netherlands Institute of Ecology (NIOO-KNAW), Wageningen, The Netherlands; 4 Present Address: The Netherlands Food and Consumer Product Safety Authority, Utrecht, The Netherlands; 5 Present Address: LPTMS, Université Paris-Sud 11, UMR 8626 CNRS, Orsay Cedex, France; 6 Present Address: Physical Chemistry and Soft Matter, Wageningen University, Wageningen, The Netherlands; 7 Present Address: Veluws College, Twello, The Netherlands; 8 Present Address: Biomedic JSC, Hanoi, Vietnam

**Keywords:** Molecular evolution, Bacterial genes

## Abstract

While synonymous mutations were long thought to be without phenotypic consequences, there is growing evidence they can affect gene expression, protein folding, and ultimately the fitness of an organism. In only a few cases have the mechanisms by which synonymous mutations affect the phenotype been elucidated. We previously identified 48 mutations in TEM-1 β-lactamase that increased resistance of *Escherichia coli* to cefotaxime, 10 of which were synonymous. To better understand the molecular mechanisms underlying the beneficial effect of these synonymous mutations, we made a series of measurements for a panel containing the 10 synonymous together with 10 non-synonymous mutations as a reference. Whereas messenger levels were unaffected, we found that total and functional TEM protein levels were higher for 5 out of 10 synonymous mutations. These observations suggest that some of these mutations act on translation or a downstream process. Similar effects were observed for some small-benefit non-synonymous mutations, suggesting a similar causal mechanism. For the synonymous mutations, we found that the cost of resistance scales with TEM protein levels. A resistance landscape for four synonymous mutations revealed strong epistasis: none of the combinations of mutations exceeded the resistance of the largest-effect mutation and there were synthetically neutral combinations. By considering combined effects of these mutations, we could infer that functional TEM protein level is a multi-dimensional phenotype. These results suggest that synonymous mutations may have beneficial effects by increasing the expression of an enzyme with low substrate activity, which may be realized via multiple, yet unknown, post-transcriptional mechanisms.

## Introduction

Synonymous mutations occur due to redundancy in the genetic code: 64 codons are available to specify 20 amino acids and stop codons. The different codons for the same amino acid were long thought to be “silent”, being functionally equivalent, and without phenotypic consequences. Universally accepted approaches in phylogenetics—such as dN/dS analysis—rest on the assumption that synonymous mutations are by-and-large selectively neutral. While such assumptions are useful first approximations, there is mounting evidence that for many organisms, some synonymous mutations are anything but silent.

One of the first strong clues that synonymous mutations might not be functionally equivalent was that codon bias varies between species and loci (Anderson and Kurland 1990; Hershberg and Petrov 2008). There have been many experimental observations of effects of synonymous mutations on phenotypes in different organisms (Carrasco et al. 2007; Lind et al. 2010; Plotkin and Kudla 2011; Agashe et al. 2012; Salari et al. 2013). Salient highlights include the strong effects of synonymous mutations on gene expression (Gustafsson et al. 2004), the implication of synonymous mutations in human disease (Kimchi-Sarfaty et al. 2007) and the finding that combinations of deleterious synonymous mutations can be lethal (Lalić and Elena 2012). Fixation of beneficial synonymous mutations during experimental evolution has also been observed (Bailey et al. 2014; Agashe et al. 2016).

There are many mechanisms by which synonymous mutations are thought to affect phenotypes (Anderson and Kurland 1990; Plotkin and Kudla 2011). Synonymous mutations could affect the structure of the messenger molecule. Low 5′ stability is thought to lead to higher rates of translation, whereas stem-loop structures throughout the open reading frame can have varying effects on translation. The incorporation of codons, which are rarely used and have a low abundance of tRNAs, or Shine-Dalgarno-like sequences (Li et al. 2012), could lower translation rates locally. Any mechanism that affects translation rates could change the amount of synthesized protein, translation accuracy, and co-translational protein folding. Finally, different rates and spectra of translation errors for different synonymous codons could affect the phenotype, resulting in deleterious or possibly even beneficial effects (e.g., a small fraction of phenotypic mutants with increased activity). In one experiment to test the effects of high rates of translation errors, non-synonymous mutations, which increased protein stability were observed rather than synonymous mutations (Bratulic et al. 2015).

TEM-1 beta-lactamase hydrolyzes the beta-lactam ring of penicillin and cephalosporin antibiotics, inactivating these therapeutic compounds and allowing bacteria to survive and grow. The TEM-1 allele was originally discovered in 1963 (Datta and Kontomichalou 1965), and found to provide resistance against penicillin and ampicillin. However, as new antibiotics were introduced into the clinic, resistant bacterial strains emerged (Medeiros 1997; Salverda et al. 2010). Resistance was often mediated by mutations in the coding region of TEM-1, allowing this resistance gene to degrade an extended spectrum of beta-lactam antibiotics, such as cefotaxime (CTX). Experimental evolution—involving repeated cycles of error-prone PCR followed by selection in the presence of antibiotics—has been used to show how resistance to CTX can evolve rapidly through a small number of non-synonymous mutations in TEM-1 (Barlow and Hall 2002). Subsequently, TEM-1 has become a widely used model system to study evolution. For example, laboratory studies employing TEM have shown the importance of epistatic interactions between mutations for determining evolutionary trajectories (Weinreich et al. 2006; Salverda et al. 2011, 2017), characterized the distribution of mutational fitness effects (Schenk et al. 2012; Jacquier et al. 2013; Firnberg et al. 2014; Stiffler et al. 2015), and measured the strength of epistasis between beneficial mutations (Schenk et al. 2013). Not only have some molecular mechanisms underlying increased resistance been elucidated, even the mechanism by which epistasis arises from structural changes has been documented in detail (Dellus-Gur et al. 2015).

For the TEM gene, not all synonymous mutations are silent. Some synonymous mutations are repeatedly found in clinical samples, such as mutation F8* that occurs in 42/179 alleles (Salverda et al. 2010). Note that we use an asterisk after the amino-acid position to denote synonymous mutations throughout the manuscript. Whether these synonymous changes are genetic “hitchhikers” or adaptive themselves because they impact resistance, is not clear. In laboratory evolution experiments with TEM, synonymous mutations are often found (Salverda et al. 2011), and sometimes contribute to higher resistance or fitness in the absence of antibiotics (van Dijk et al. 2017) or occur in more replica populations than expected by chance (Salverda 2008). In one study that systematically explored deleterious mutational effects, there was little evidence for strong mutational effects on resistance by synonymous mutations (Jacquier et al. 2013). However, in another study where fitness was measured, there was clear evidence for fitness effects of synonymous mutations (Firnberg et al. 2014). These effects appear to be related to 5′ folding of the messenger, and result in differences in TEM protein expression levels, as also shown previously (Zalucki et al. 2008). In a systematic screen for beneficial single-nucleotide mutations for a novel antibiotic (CTX), we found that 10 out of 48 identified beneficial mutations were synonymous (Schenk et al. 2012). These mutations had relatively small effects on resistance, leading to a 2.3-fold increase at most, whereas the most beneficial non-synonymous mutation gave a 27-fold increase in resistance. Interestingly, 3 of these synonymous mutations have also been observed in clinical isolates (F8*, R9*, and E89*), while 2 (R9* and A17*) have also been observed during laboratory evolution (Salverda et al. 2011). The mechanism(s) by which these 10 synonymous mutations affect resistance is however unclear. Given previous results (Zalucki et al. 2008; Firnberg et al. 2014), one likely route by which these mutations might affect resistance is by altering TEM expression.

Whereas Wright formulated the concept of the fitness landscape, it has recently become possible to analyze empirical fitness landscapes by systematically generating all combinations of mutations and then measuring fitness for the resulting genotypes (de Visser and Krug 2014). These studies often have revealed strong epistasis, leading to rugged landscapes with a limited number of accessible mutational trajectories to a global optimum. Whilst empirical fitness landscapes are important tools for understanding and predicting evolutionary dynamics, they can also help to elucidate mechanistic features of mutations. For example, assuming that mutations act additively on a set of underlying phenotypes that in turn determine fitness, the dimensionality of these phenotypes can be inferred by analyzing the interactions between mutations. This approach has been used to identify one-dimensional phenotypes that can overshoot a fitness optimum (Rokyta et al. 2011) or more complex multi-dimensional phenotypes (Schenk et al. 2013; Schoustra et al. 2016).

Here we set out to identify the mechanism by which the 10 synonymous mutations in TEM identified by Schenk et al. (2012) increase resistance to CTX. First, for a panel consisting of the 10 synonymous mutations, 10 non-synonymous mutations, and the ancestral TEM-1 allele, in *Escherichia coli* we measured, (1) TEM messenger levels, (2) total (soluble) TEM protein levels, (3) (soluble) functional TEM protein levels, (4) competitive fitness, and (5) induction of selected stress responses. Second, mutants carrying all combinations of four of these synonymous mutations were constructed and their resistance was measured to determine the strength of epistatic interactions between these mutations and shed light on the dimensionality of the phenotypes and thereby the diversity of mechanisms that contribute to increased resistance.

## Materials and methods

### Bacterial strains and plasmids

All experiments were carried out with *E. coli* DH5αE. TEM alleles were ligated into the pACSE3 plasmid (Barlow and Hall 2002), which contains a tetracycline resistance cassette, to render the pACTEM plasmid and electrotransformed in *E*. *coli* as previously described (Schenk et al. 2012). TEM is then under the control of the pTac promotor, and to induce expression 50 μM isopropyl β-d-1-thiogalactopyranoside (IPTG) is added. The TEM-1 sequence is identical to that present in pBR322 (GenBank accession J01749).

### Media

As in previous work on these mutations (Schenk et al. 2012, 2013), Lysogeny broth (LB) medium is 10 g/L trypticase peptone, 5 g/L yeast extract, and 5 g/L NaCl. SOC medium consists of 20 g/L bacto-tryptone and 5 g/L yeast extract, to which is added 10 mM NaCl, 2.5 mM KCl, 10 mM MgCl_2_, and 20 mM glucose. For plates, 15 g/L agar was added.

### Construction of mutants

The QuikChange Site-Directed Mutagenesis Kit (Stratagene) was used to construct TEM alleles for the synonymous mutation landscape of mutations R9*, A17*, G87*, and E89*. After construction, the plasmid was transformed into new *E*. *coli* cells and the TEM locus was sequenced again to verify the identity of the allele.

ET recombination (Zhang et al. 1998; Muyrers et al. 1999) was used to obtain a ΔrpoS *E*. *coli* strain. This strain was constructed to test whether this stress pathway plays a role in mediating higher resistance for some of the synonymous mutations. The KanR2 resistance cassette was PCR-amplified with primers that introduced 50 bp homologous arms: 5′-AGGCTTTTGCTTGAATGTTCCGTCAAGGGATC- ACGGGTAGGAGCCACCTAAATC-CTGATGTTACATTGCAC-3′ and 5′-ACAGAAAAGGC CAGCCTCGCTTGAGACTGGCCTTTCTGACAGATGCTTACCTCTGCCAGTGTTACAACCA-3′. Following transformation and selection of recombinants with 50 μg/mL kanamycin, the rpoS locus was sequenced to verify correct deletion of the gene.

### TEM messenger levels and the induction of *E*. *coli* stress responses

To measure TEM messenger levels, clones were revived in 2 mL LB with 15 μg/mL tetracycline and incubated at 37 °C with agitation at 250 r.p.m. (New Brunswick Scientific 12500 series incubator). We then diluted the overnight culture 1:100 in 25 mL LB with 15 μg/mL tetracycline, and incubated as before. After 2 h, TEM expression was induced by the addition of IPTG to 50 μM. The cultures were then incubated for at least 1 h longer at 37 °C. OD_600_ measurements were made after 60 and 90 min to ensure that all cultures were harvested when the OD_600_ was between 0.4 and 0.6, which occurred for all mutants within 90 min. Cultures were then placed on ice, and divided into three batches: 10 mL for total RNA extraction; 10 mL for protein extraction; and 1 mL was stored for plasmid DNA extraction to ensure plasmid integrity. To check plasmid integrity, we extracted plasmid DNA and resolved it on a 1% agarose gel.

To proceed with RNA extraction, cells were centrifuged for 5 min at 3500 × *g* at 4 °C, resuspended in 100 μL TE (Tris-HCL 10 mM, diaminoethane tetraacetic acid) with 1 mg/ml lysozyme, and incubated at 37 °C with mild agitation for 10 min. We then proceeded with the NucleoSpin RNA kit Mini (Machery Nagel), using the protocol for RNA extraction from bacteria provided in the manual (the next step is the addition of 350 μL Buffer RA1 [from the kit] with 20 mM dithiothreitol [DTT]). RNA was stored at −80 °C.

We then performed reverse-transcription quantitative PCR (RT-qPCR) to quantify TEM transcript levels, and measure the induction of *E*. *coli* stress responses. For reverse transcription, iScript (Bio-Rad)—which includes random hexamer primers in the master mix—was used following the manufacturer’s instructions. qPCR was performed using Bio-Rad iB SYBR Green Supermix in a 20 μL total reaction volume with 250 pM forward and reverse primers, run on the Bio-Rad CFX Connect system. All primers used for qPCR are given in Table S1 (Supplementary online material). Standard curves were performed using purified PCR products, diluted to known concentrations of template copies ranging from 10^8^ to 10^2^ copies/μl. We measured copy numbers of TEM, *rpoS*, *relA*, *spoT*, *sulA*, and *hipA*. To normalize expression levels, we measured expression levels of two stably expressed housekeeping genes (*rrsA* and *cysG*) (Zhou et al. 2011; Peng et al. 2014), and the constitutively active tetracycline resistance gene on pACTEM (*TcR*). We divided the copy numbers of the gene of interest by the geometric mean of the three normalization genes.

### Total TEM protein levels

To measure total TEM protein levels, we first extracted protein from the 10 mL of culture allocated for this purpose (see Measurement of TEM messenger levels above). This culture was centrifuged for 5 min at 3500 × *g* at 4 °C and resuspended in 1 mL sonification buffer (50 mM Tris-HCl [pH = 8.0], 0.5 M NaCl, and 10% glycerol). Afterwards 142 μL protease inhibitor solution (1 tablet of cOmplete Mini Protease Inhibitor Cocktail in 1.5 mL sonification buffer) was added, followed by 5 µL 1 M DTT. The sample was then sonificated twice for 5 s. The homogenate was then centrifuged at 21 000 relative centrifugal force for 10 min at 4 °C. Aliquots of the supernatant were then stored at –20 °C. Total protein was subsequently measured using the Bradford method. We then ran 5 μg total protein of these samples on 12% acrylamide SDS-polyacrylamide gel electrophoresis gel, and transferred the protein to a nitrocellulose membrane by wet electroblotting. Ponceau staining was then used to visualize the total protein on the blots. After removing the Ponceau staining, TEM was detected by western blot with a mouse monoclonal antibody against TEM-1 (AbCam antibody 122251), using a goat anti-mouse horseradish peroxidase as a secondary antibody (Bio-Rad 170-6516). On every blot we included a reference sample of TEM-1, obtained from aliquots of a stationary phase TEM culture. We used Bio-Rad Image Lab 2.01 software to determine the TEM signal (TEM band intensity in the Western) and the total protein content (the sum of all band intensities detected by the Ponceau staining), and the quotient is the relative TEM expression. This ratio was then normalized by the TEM-1 reference signal, to account for any differences in signal intensity due to minor experimental variation.

### Functional TEM protein levels

To quantify the level of functional TEM protein, we developed an assay based on the hydrolysis of nitrocefin. Nitrocefin is a chromogenic cephalosporin analogous to ampicillin without antibiotic activity. Under the assumption that our TEM mutations do not affect catalytic activity against nitrocefin (which seems likely particularly for the 10 synonymous mutations), nitrocefin hydrolysis rates effectively measure functional TEM protein levels. As a standard curve, we used extracted protein from a TEM-1 stationary phase culture (soluble fraction), and made a seven-step twofold dilution series with total protein concentrations ranging from 3.3 μg/mL to 52 ng/μL in phosphate buffer (pH = 7.0). Unknown protein samples for which activity was to be determined were diluted to a concentration of 180 ng/μL. All samples were then assigned a random position on a 96-well plate, and 300 μl diluted sample was added to the plate, while being chilled on ice. Rehydrated nitrocefin was then rapidly added to each sample to a concentration of 2 mg/mL. In pilot experiments, having a low amount of beta-lactamase (i.e., highly diluted samples) and a high concentration of nitrocefin was essential for quantification, as beta-lactamase rapidly degrades nitrocefin. Upon adding nitrocefin, the plate was immediately placed in a Victor^3^ plate reader (Perkin-Elmer), incubated at room temperature (21 °C) and the OD_490_ was measured every minute for 30 min.

A linear model was then fitted to the square-root-transformed OD_490_ data for the standard curve, and the data for the time point with the highest coefficient of determination were used for subsequent analysis. This procedure typically rendered maximum *r*^*2*^ values > 0.99 after 15–20 min of incubation, and the fitted standard curve was then used to calculate relative concentrations for unknown samples.

### Bulk competitions

To assess competitive fitness of the mutant TEM alleles, TEM-1 and the 20 beneficial alleles were grown up overnight, subcultured, and a mixture was made of all alleles at equal frequencies, using estimates of cell densities from OD_600_ values. Bulk competitions were then performed in 5 mL liquid cultures, at two starting densities: 5 × 10^4^ and 5 × 10^6^ cells/mL. LB with 50 µM IPTG and no CTX was used, and the cultures were not agitated. A volume of 50 µL culture was sampled at the start of the competitions and 8 h post inoculation, and the frequency of TEM alleles was then determined by Illumina HiSeq 2500 paired-end deep sequencing of the PCR-amplified TEM allele (Salverda et al. 2017). The frequency of the unique single-nucleotide polymorphism contained in each allele was used to estimate allele frequency, and we combined estimates of the selection rate constants (Salverda et al. 2017) from the low- and high-density data. Assuming that the frequency of TEM-1 is one minus the sum of all other allele frequencies did not allow reliable estimates of the fitness of the TEM-1 allele. Therefore, to make comparisons to the wild-type fitness, we also performed pairwise competitions against TEM-1 with G87*, E89*, and G238S, using a starting density of 5 × 10^6^ cells/mL and otherwise under the same conditions and with the same readout assay as described above.

### Statistical analyses of panel data

For the analysis of the experimental results for the panel of 20 beneficial alleles (stress response indicators and TEM expression data), we first used Welch’s test to test for overall significant differences between treatments (alleles), as assumptions on homoscedasticity for analysis of variance were not met. As a post hoc test, we performed pairwise *t-*tests between mutant alleles and TEM-1. For all independent sample *t-*tests, we assumed constant variance only if a Levene test gave an insignificant outcome. We used the Benjamini-Hochberg procedure (Benjamini and Hochberg 1995) to keep the false discovery rate at 1/20 for these pairwise *t-*tests.

### Measurement of resistance to CTX

To measure CTX resistance of TEM alleles, we determined the inhibitory concentration killing 99.99% (IC_99.99_, Schenk et al. 2012). For this assay, un-induced (i.e., no IPTG) exponential phase cells are plated out at different cellular densities (200, 2 × 10^4^, and 2 × 10^6^ cells/6 cm plate) on plates with solid medium supplemented with 15 μg/mL tetracycline, 50 μM IPTG, and different CTX concentrations (0.015625–0.5 μg/mL CTX, by twofold increments). Colonies are counted after 40 h of incubation at 37 °C.

In previous work, we determined the IC_99.99_ antibiotic concentration by linear interpolation between the two concentrations and plate densities straddling this point. Here we refined this analysis, by fitting a dose response model to all the data using a maximum likelihood method, and inferring the IC_99.99_ from the fitted model. We used a general dose response model: $S_C = \alpha e^{ - \beta C^\gamma }$, where *S* is survival, *C* is the concentration of CTX, and model constants *α*, *β*, and *γ* need to be estimated from the data. This model was fitted to the data using a stochastic hill-climbing algorithm using a custom R (R Core Team 2016) script. It was assumed that the observed colony counts follow a Poisson distribution with a mean *λS*_*C*_, where *λ* is the expected number of colonies based on control plates with no CTX.

## Results

**Fig. 1:**
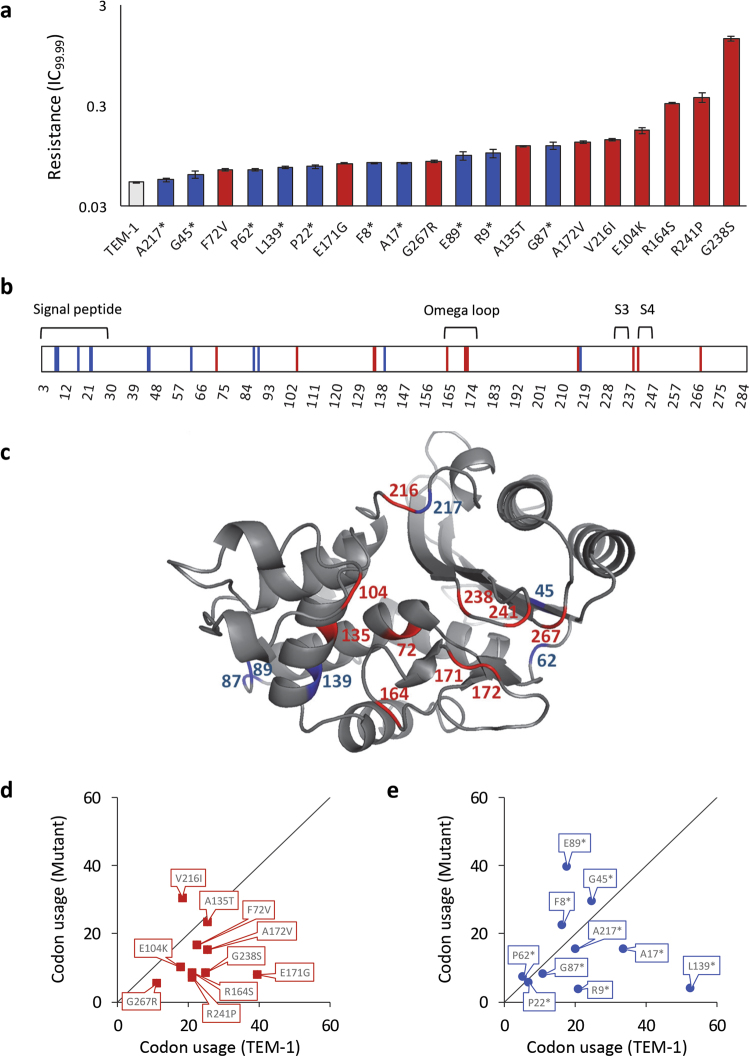
An overview of the panel of synonymous and non-synonymous mutations. For all panels, blue data correspond to the synonymous mutations and red to the non-synonymous mutations. In **a**, resistance against CTX in μg/mL [IC_99.99_] is given for the whole panel of TEM alleles. Error bars are standard errors of the mean (*N* = 3). In **b**, the location of the mutations and key features of TEM are given, with numbers indicating amino-acid positions. In **c**, the mutations present in the mature TEM protein are shown (four synonymous mutations occur in the signal peptide), with numbers indicating amino-acid positions. In **d** and **e**, the codon usage for original TEM sequence (*x*-axis) and the mutants (*y*-axis) is given, with units codons per 1000 codon positions. **d** Codon use for the non-synonymous mutations, and **e** codon use for the synonymous mutations. Although some mutations lead to clear differences in codon usage, there is no systematic pattern for either mutation class

To better understand the mechanism by which synonymous mutations in TEM-1 increase resistance to CTX, we performed a number of measurements on a panel of 21 TEM alleles: the wild-type TEM-1 allele; 10 single-nucleotide synonymous mutations (i.e., all available ones); and 10 single-nucleotide non-synonymous mutations (selected for their different effect size on CTX resistance from the 38 available mutations; Table S2) previously identified (Schenk et al. 2012). For the full set of 48 beneficial mutations previously identified, synonymous mutations have a smaller effect on resistance than non-synonymous mutations (Schenk et al. 2012). Within the panel of 21 alleles used here, the synonymous mutations also have smaller effect sizes than the non-synonymous mutations (Fig. 1a; Mann-Whitney test: *U* = 83, *n*_1_ = *n*_2_ = 10, *P* = 0.011). The synonymous mutations are mostly near the 5′ end of the gene (Fig. 1b), whereas many of the non-synonymous mutations are located near the omega loop and between the S3 and S4 domains (Fig. 1c) (Schenk et al. 2012).

We first considered whether there were any systematic changes in codon abundance for the panel of mutated TEM alleles, by comparing the frequency of the original and modified codons in the *E*. *coli* genome (Fig. 1d, e). For the non-synonymous mutations, most mutations switch toward rarer codons (below the diagonal in Fig. 1d), while only one mutation introduces a more abundant codon (above the diagonal in Fig. 1d). Hence the non-synonymous mutations cause on average a significant change toward rarer codons (one-sample *t*-test of the ratio of original codon usage divided by new codon usage vs. a value of 1: *t*_9_ = −2.829, *P* = 0.020). For the synonymous mutations, there were no clear trends as mutations switched to both more rare and more abundant codons (one-sample *t-*test: *t*_9_ = −0.499, *P* = 0.630), nor was there a difference in codon abundance between the synonymous and non-synonymous populations (independent samples *t-*test: *t*_18_ = −1.129, *P* = 0.274). Empirically supported indices of the effects of codon usage on expression can be superior predictors of expression levels compared to codon frequency in the genome (Boël et al. 2016). We considered the predictions of one empirically supported and tested index, which includes all codons, the codon influence as determined by single-parameter binary logistic regression (Boël et al. 2016). Using this index we found effects for 2 out of 10 non-synonymous mutations, and 7 out of 10 synonymous mutations. For both kinds of mutations, codons that significantly favored or disfavored high expression were substituted (Table S3). Whilst half of the synonymous mutations (5 out of 10) are predicted to have positive effects on expression levels according to this codon influence index, 2 synonymous mutations predicted the exact opposite effect. Overall, the codon usage results therefore fail to reveal any clear trends.

We then considered the stability of RNA secondary structures for the panel of TEM alleles (Fig. S1), using RNAfold 2.2.4 (Lorenz et al. 2011) predictions for a 45 bp sliding window. For the mean or maximally deviating value from TEM-1, neither the stability of the synonymous (one-sample *t*-test on Δ*G* values; means: *t*_9_ = −1.975, *P* = 0.080; maximally deviating: *t*_9_ = −0.973, *P* = 0.356) nor the non-synonymous (means: *t*_9_ = −0.663, *P* = 0.524; maximally deviating: *t*_9_ = −1.507, *P* = 0.166) mutations differed significantly from TEM-1, and there was also not a significant difference between these groups (independent samples *t*-test: means: *t*_18_ = 1.267, *P* = 0.221; maximally deviating: *t*_18_ = −0.202, *P* = 0.842). Since the codon usage and RNA stability results gave no clues as to what mechanism might increase resistance, we made a series of measurements—focusing on TEM expression—to find clues to elucidate this mechanism.

The P22* allele was excluded from all subsequent analyses, as we repeatedly detected deletions in the pACTEM plasmid carrying the P22* allele and our expression measurements for this allele are therefore unreliable. The pACTEM plasmid has an identical 185 bp sequence that occurs both up- and downstream of the TEM and *lacI* genes, leading to stability issues in the absence of selection for beta-lactamase activity.

### No clear effect on TEM messenger level

**Fig. 2:**
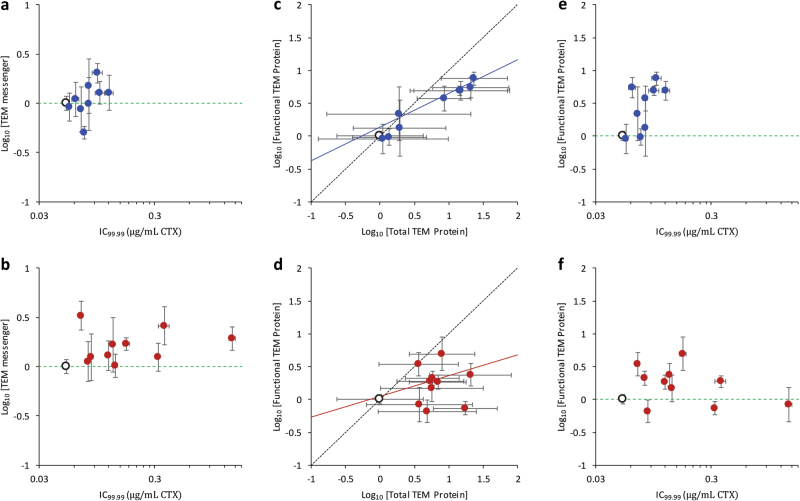
For all panels, measurements for mutants (full circles) are given relative to the TEM-1 allele (open circle), and error bars represent the standard error of the mean (*N* = 3). The relationship between CTX resistance (*x*-axis) and relative TEM messenger level (*y*-axis) is given for the synonymous (**a**) and non-synonymous mutants (**b**). The dotted green line indicates TEM-1 expression levels in **a**, **b**, **e**, and **f**. There does not appear to be a relationship between messenger levels and resistance for either mutation type. The relationship between total (*x*-axis) and functional TEM protein level (*y*-axis) is given, for the synonymous TEM mutants (**c**) and non-synonymous mutants (**d**). The dotted line is *y* = *x*, whereas the solid line is the model-2 regression. For the synonymous mutations, there is a clear relationship between total TEM protein level and measured activity (95% confidence interval of slope: 0.396–0.646), suggesting increased total protein levels correspond well with higher functional protein levels. For the non-synonymous mutations, the relationship is not as evident (95% confidence interval of slope: 0.006–0.690), although some mutations appear to have increases in total and functional protein levels. The relationship between CTX resistance (*x*-axis) and relative functional TEM protein level (*y*-axis) is given for the synonymous (**e**) and non-synonymous mutants (**f**). The dotted line indicates TEM-1 functional protein levels. For the synonymous mutations, the most resistant alleles have large increases in functional TEM protein levels, whereas some small- and intermediate-level non-synonymous mutations do show increases in functional TEM protein levels similar to those for the synonymous mutations

To test for transcription-level effects for our panel of beneficial mutations, we measured TEM messenger RNA levels by RT-qPCR. Different TEM alleles appear to show significant differences in TEM messenger level (Table S4), although a pairwise comparison vs. TEM-1 shows no significant differences after correcting for multiple testing (Fig. S2b). However, L139* was almost significant in the Benjamini-Hochberg procedure (threshold *P* value: 1/20 × 0.05 = 0.0025; *t*-test result: *t*_4_ = 6.565, *P* = 0.0028), suggesting this decrease in TEM messenger level might be real. Although overall there is variation in TEM messenger levels between alleles, there is not a statistically significant relationship between messenger levels and resistance for either synonymous or non-synonymous mutations (Fig. 2a, b; Spearman rank correlation, non-synonymous mutations: *ρ* = 0.224, *N* = 10, *P* = 0.533; synonymous mutations: *ρ* = 0.636, *N* = 9, *P* = 0.066). We therefore discarded the idea that the synonymous mutations increase TEM resistance by bolstering TEM transcript levels.

### Synonymous and non-synonymous mutations increase total and functional TEM protein levels

We then measured TEM protein levels with a western blot-based assay (Fig. S2C). Given the high levels of variation between experimental replicates, there were no statistically significant differences overall (Table S4) and in post hoc pairwise comparisons of protein levels (Fig. S2C). There is not a statistically significant relationship between total TEM protein levels and resistance for either synonymous or non-synonymous mutations (Spearman rank correlation, non-synonymous mutations: *ρ* = 0.139, *N* = 10, *P* = 0.701; synonymous mutations: *ρ* = 0.594, *N* = 9, *P* = 0.092). However, the data do suggest that some mutations, especially those with intermediate levels of CTX resistance, might be affecting TEM protein levels.

Since the western blot assay for measuring TEM protein levels introduced large measurement errors and measured all protein, functional and non-functional, a more direct measure of functional protein levels was desirable. We therefore measured quantitatively the enzymatic breakdown rate of extracted total protein samples on the chromogenic cephalosporin nitrocefin. Since we expected no or small pleiotropic effects from most of our mutations with increased CTX resistance on nitrocefin catalytic activity, this assay effectively quantified the functional TEM protein levels of the mutants. Overall, we found significant variation among TEM alleles on nitrocefin activity (Table S4), and post hoc pairwise tests with a correction for multiple comparisons showed increased functional protein levels for G45*, E171G, A17*, E89*, R9*, G87*, and R241P alleles (Fig. S2D). For the synonymous mutations, we found a high correlation between total TEM protein levels as measured by western blot and functional TEM protein as measured by hydrolysis of nitrocefin (Fig. 2c, Spearman rank correlation: *ρ* = 0.983, *N* = 10, *P* < 0.001). Some non-synonymous mutations are known to strongly affect protein structure and this might affect activity on nitrocefin through changes in catalytic activity instead of changes in expression. We therefore anticipated, and found, no correlation between total TEM protein levels and nitrocefin hydrolysis rates for the non-synonymous mutations (Fig. 2d, Spearman rank correlation: *ρ* = 0.176, *N* = 33, *P* = 0.627). Some of the small- and intermediate-size non-synonymous mutations do, however, show similar increases in total TEM protein level and nitrocefin breakdown rate, suggesting that also some non-synonymous mutations show their benefit via increased functional protein expression levels.

These results, therefore, suggest that some of the synonymous mutations confer increased resistance through increased levels of functional TEM protein (Fig. 2e), despite low activity against CTX, which in turn results from a process downstream of transcription. For some of the synonymous mutations we have investigated—including the clinically relevant F8*—we found no increase in TEM protein levels, suggesting there are other, as of yet unknown mechanisms by which some of these mutations act. For example, F72V messenger, total protein, and functional protein levels were not significantly higher than for TEM-1 after correcting for multiple comparisons, but all of these levels consistently appear to be 3.5-fold higher than TEM-1. These observations suggest that F72V may have increased transcript levels, and that this results in an increase in TEM protein level.

### No observable effects of TEM allele on *E*. *coli* stress responses

As a possible alternative mechanism for the observed benefit of the synonymous mutations, we considered whether the TEM alleles in our panel might have different effects on the level of induction of *E*. *coli* stress responses, by measuring messenger RNA levels of some known stress indicator genes. We hypothesized that if a mutation leads to a large increase in the stalling of translation for the—in our setup—highly transcribed TEM, or leads to the depletion of a particular tRNA, it might contribute to the increased triggering of stress responses, and hereby possibly increased CTX resistance. However, we did not find any evidence that stress responses play a role in increasing resistance (see Appendix 1 and Figs. S3 and S4).

### Functional TEM levels are inversely correlated with competitive fitness in the absence of CTX for the synonymous mutations

**Fig. 3:**
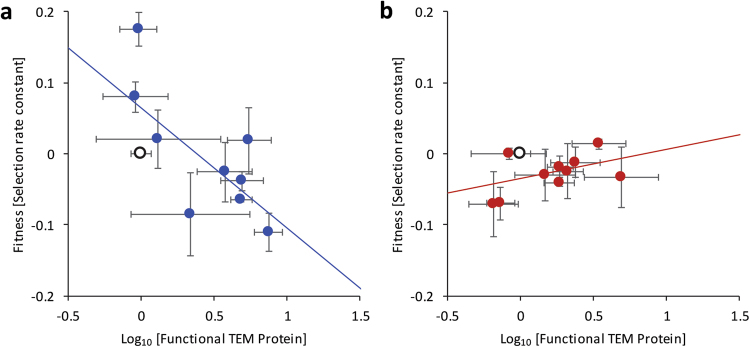
For both panels, measurements for mutants (full circles) are given relative to the TEM-1 allele (open circle), error bars represent the standard error of the mean (*N* = 3), and the solid line is a model-2 regression. The relationship functional TEM protein level (*x*-axis) and fitness in the absence of CTX is given, for the synonymous TEM mutants (**a**) and non-synonymous mutants (**b**). For the synonymous mutations, there is clear inverse relationship between functional TEM protein levels and fitness (95% confidence interval of slope: −0.315 to −0.030). Note that some mutations without increased expression have high fitness, in particular L139*. For the non-synonymous mutations, there is no relationship between functional TEM protein levels and fitness (95% confidence interval of slope: −0.026 to 0.109)

Next, we performed bulk competitions in the absence of antibiotics to estimate fitness effects of the alleles, in order to test for costs associated with high functional TEM protein levels (Fig. 3). The fitness of non-synonymous and synonymous alleles was not significantly different (*t*-test, equal variances not assumed: *t* = 0.860, d.f. = 9.370, *P* = 0.402), although there was more variation in fitness for the synonymous mutations than for the non-synonymous mutations (Levene test: *F*_1,17_ = 7.766, *P* = 0.013). When we compared functional TEM protein levels to fitness, there was a significant negative correlation for the synonymous mutations (Fig. 3a; Spearman rank correlation: *ρ* = −0.700, *N* = 9, *P* = 0.036), whereas there was not a significant correlation for the non-synonymous mutations (Fig. 3b; Spearman rank correlation: *ρ* = 0.515, *N* = 10, *P* = 0.128). These results allow us to draw two conclusions. First, high functional TEM protein levels are associated with low fitness for the synonymous mutations, while their gains in resistance are only modest. Whilst these mutations could be enriched upon low-cellular-density selection for resistance (Schenk et al. 2012), they are unlikely to be competitive with non-synonymous mutations that can confer higher resistance without this cost, or when there are interactions between resistant and susceptible strains (Yurtsev et al. 2013). Second, some synonymous mutations appear to increase fitness in the absence of antibiotics, the most striking case being L139*. Although all mutations in the panel have increased resistance to CTX (Schenk et al. 2012), these data suggest that the benefits of some synonymous mutations are not exclusively linked to increased resistance per se, but rather to improved competitive ability.

### Interactions between synonymous mutations

**Fig. 4:**
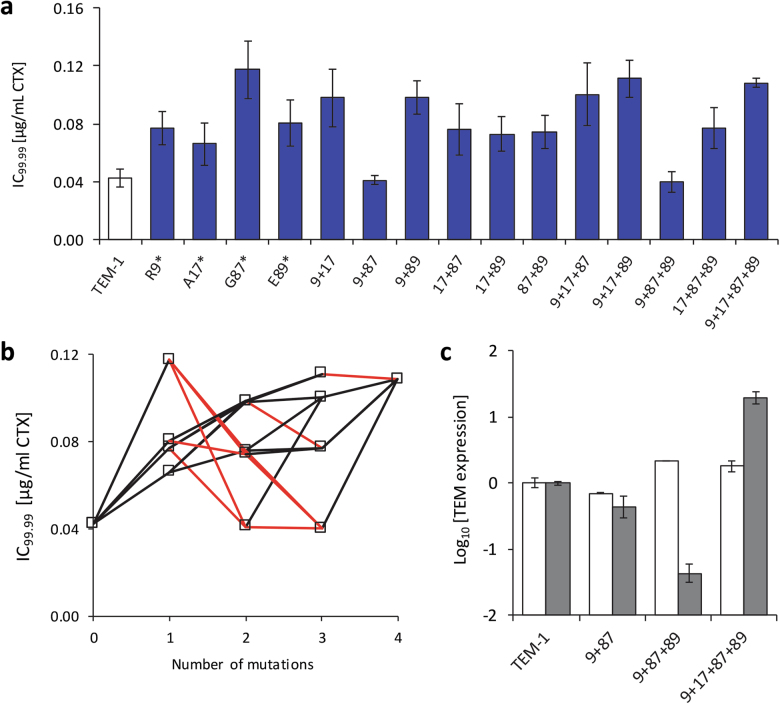
Resistance landscape of four beneficial synonymous mutations: R9*, A17*, G87*, and E89*. All error bars indicate the standard error of the mean (*N* = 3 for **a**, *N* = 2 for **c**). In **a**, resistance to CTX (IC_99.99_) is shown for all alleles in the fitness landscape. In **b**, the same data are represented, but with the number of mutations on the *x*-axis and resistance on the *y*-axis. If the resistance increases as an extra mutation is added, the connecting line is black, whereas if resistance decreases the line is red. In **c**, TEM expression data for a number of selected alleles are given. TEM messenger levels are indicated by white bars, whereas TEM protein levels are indicated by gray bars. For the low-resistance alleles R9*/G87* and R9*/G87*/E89*, TEM protein expression levels appear to drop, whereas protein levels are high for the quadruple mutant with high resistance

We constructed and analyzed mutants with all combinations of four synonymous mutations (R9*, A17*, G87*, and E89*), to test how these mutations interact with each other and to help elucidate the underlying mechanisms that increase resistance to CTX. We chose to construct this “resistance landscape” for the four largest-effect mutations, but used A17* (fifth largest) instead of F8* (fourth largest), because F8* alters the codon adjacent to R9*. Note that all of these mutations increase total and functional TEM protein levels. When we measured the IC_99.99_ of these mutants, we found overall significant differences in resistance between alleles (Welch’s test: test statistic = 35.713, d.f. = 15, 11.937, *P* < 0.001). None of the alleles had higher resistance than G87* (Fig. 4), the largest-effect single mutation. For the combination of R9* and G87*, resistance falls to the same level as TEM-1, although the addition of the A17* mutation then mitigates this strong interaction. The quadruple mutant has a resistance similar to G87*. For the two low-resistance combinations of mutations (R9*/G87* and R9*/G87*/E89*), as well as for the quadruple mutant, we measured TEM expression at the messenger and total protein levels (Fig. 4c). Whereas messenger levels were reasonably constant, we found low TEM protein levels for the low-resistant double and triple mutants, while for the quadruple mutant with relatively high resistance, high TEM protein levels were found (Fig. 4c). Thus for these alleles, resistance again correlates well with protein expression levels.

### Quantifying epistasis between synonymous mutations

**Fig. 5:**
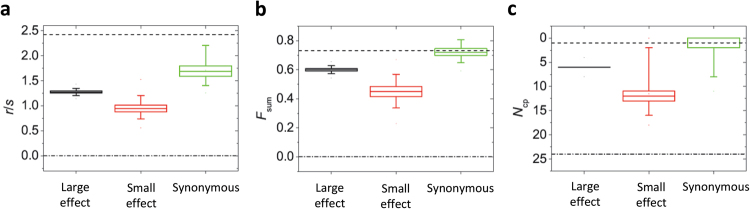
Three measures of ruggedness for the synonymous resistance landscape, with results for large-effect and small-effect non-synonymous resistance landscapes (Schenk et al. 2013) for comparative purposes. The measures are the roughness-to-slope ratio *r*/*s* (**a**), the fraction of total variation explained by epistatic interactions, *F*_sum_ (**b**), and the number of paths to the global resistance maximum from its antipode along which resistance increases monotonically, *N*_cp_ (**c**). The median is indicated by the central line, the box indicates the interquartile range and the error bars represent the 1st and 99th percentile, based on resampling the data. The maximally rugged and additive expectations are given by the dashed and dashed-dotted lines, respectively

In order to quantify the epistatic interactions among the four synonymous mutations, we carried out two different types of analyses. First, we computed a set of global measures of fitness landscape ruggedness described in Szendro et al. (2013) and applied to two sets of four non-synonymous resistance-increasing mutations in TEM-1 (Schenk et al. 2013). In this previous study, the empirical resistance landscape was studied for two sets of mutations, with either large or small effects on resistance to CTX. These measures comprise: (i) the roughness-to-slope ratio *r*/*s*; (ii) the fraction of total variation explained by epistatic interactions, *F*_sum_; and (iii) the number of paths to the global resistance maximum from its antipode along which resistance increases monotonically at each mutational step, *N*_cp_. According to all three measures, the synonymous landscape is more rugged than the two non-synonymous mutation landscapes (Fig. 5). This is surprising because the synonymous mutations necessarily act on a much more restricted set of phenotypes and therefore a lower complexity of interactions might have been expected. Note, however, that recent theoretical work on Fisher’s geometric model has shown that the complexity of epistatic interactions does not need to correlate with the number of phenotypes under selection (Blanquart et al. 2014; Hwang et al. 2017).

Further insight into the origin of these pronounced interactions can be gained by decomposing the resistance landscape into contributions from different subsets of mutations. For this purpose we encode each genotype by a binary sequence *σ* = (*σ*_1_, *σ*_2_, *σ*_3_, *σ*_4_), where *σ*_*i*_ = 1 (*σ*_*i*_ = −1) stands for the TEM-1 (mutant) allele at position *i*. The resistance of a genotype can then be written in the form

1 $$\begin{array}{l}r\left( \sigma \right) = a^{\left( 0 \right)} + \mathop {\sum}\limits_{i = 1}^4 {a_i^{\left( 1 \right)}\sigma _i} + \mathop {\sum}\limits_{i,j} {a_{ij}^{\left( 2 \right)}\sigma _i\sigma _j} + \mathop {\sum}\limits_{i,j,k} {a_{ijk}^{\left( 3 \right)}\sigma _i\sigma _j\sigma _k} \cr + a_{1234}^{\left( 4 \right)}\sigma _1\sigma _2\sigma _3\sigma _4\end{array}$$

where the second and third sum on the right-hand side are performed over all six pairs and four triples of mutations, respectively. Equation (1) is known as the Fourier-Walsh decomposition of the landscape (Szendro et al. 2013; Neidhart et al. 2013; Weinreich et al. 2013; Poelwijk et al. 2016). The Fourier spectrum *F*(*n*) of the landscape is obtained by summing the squared coefficients $\left| {a^{\left( n \right)}} \right|^2$ for each order *n* and normalizing the result in such a way that $\mathop {\sum }\limits_{n = 1}^4 F\left( n \right) = 1$ (Neidhart et al. 2013). The global epistasis measure *F*_sum_ displayed in Fig. 5 is then given by $F_{{\mathrm{sum}}} = \mathop {\sum }\limits_{n = 2}^4 F\left( n \right) = 1 - F\left( 1 \right)$.

Fig. S5A displays the Fourier spectrum of the synonymous landscape in comparison to the two non-synonymous mutation landscapes previously studied (Schenk et al. 2013). Apart from being the most rugged of the three, the synonymous landscape is distinguished by having the lowest contribution of linear effects *F*(1) and a much larger contribution from higher-order epistatic interactions (*k* > 2) compared to the non-synonymous landscapes. The implications of higher-order epistatic interactions have been the subject of considerable recent interest (Weinreich et al. 2013; Sailer and Harms 2017a, b), and our results suggest that they may be particularly prevalent among synonymous mutations.

The Fourier-Walsh expansion (Eq. 1) provides a suitable framework for decomposing epistatic effects into contributions from different orders of interactions, but it is less informative for identifying effects of specific groups of mutants. This is because the expansion coefficients in Eq. 1 are proportional to epistatic effects averaged over all genetic backgrounds (Poelwijk et al. 2016). For example, *a*^(0)^/16 is the resistance averaged over all 2^*L*^ = 16 genotypes, and $- a_i^{\left( 1 \right)}{\mathrm{/}}8$ is the mutational effect of mutation *i* averaged over all 2^3^ = 8 combinations of the remaining three positions. A more direct representation of epistatic effects associated with particular sets of mutations is obtained by encoding genotypes in binary sequences *τ* = (*τ*_1_, *τ*_2_, *τ*_3_, *τ*_4_), where *τ*_i_ = 0 (1) stands for the absence (presence) of the *i*th mutation. The corresponding decomposition

2 $$\begin{array}{l}r\left( \tau \right) = b^{\left( 0 \right)} + \mathop {\sum }\limits_{i = 1}^4 b_i^{\left( 1 \right)}\tau _i + \mathop {\sum }\limits_{i,j} b_{ij}^{\left( 2 \right)}\tau _i\tau _j + \mathop {\sum }\limits_{i,j,k} b_{ijk}^{\left( 3 \right)}\tau _i\tau _j\tau _k\cr + b_{1234}^{\left( 4 \right)}\tau _1\tau _2\tau _3\tau _4,\end{array}$$

is sometimes referred to as a Taylor expansion, because in its truncated form it provides an approximation to the local fitness landscape around the reference sequence *τ* = (0, 0, 0, 0) that becomes increasingly accurate as higher-order terms are included (Poelwijk et al. 2016). In this expansion *b*^(0)^ is the resistance of the TEM-1 reference sequence, $b_i^{\left( 1 \right)}$ is the effect of mutation *i* on the TEM-1 background, $b_{ij}^{\left( 2 \right)}$ is the pairwise epistasis between mutations *i* and *j* on the same background, and so on. In Fig. S5B we show the mean squared Taylor coefficients of the three landscapes as a function of order, with each data set normalized to the linear effect size. The prevalence of higher-order effects in the synonymous landscape is even more prominent than for the Fourier spectrum displayed in Fig. S5A.

A conspicuous pattern that appears to be specific to the synonymous landscape is found in the signs of the Taylor coefficients of different orders (Table S5): all coefficients of odd order (*k* = 1 and 3) are positive, and those of even order (*k* = 2 and 4) are invariably negative. While the fact that all linear effects (*k* = 1) are positive follows trivially from the selection of resistance-enhancing mutations, the alternating signs for *k* ≥ 2 are unexpected. We speculate that they may be related to another striking feature of the synonymous landscape, viz. the appearance of “synthetically neutral” combinations such as R9*/G87* and R9*/G87*/E89* that have resistance levels indistinguishable from TEM-1. To see this, note that in order for the combination of two resistance mutations *i* and *j* with $b_{i,j}^{(1)} > 0$ to have a null effect, it is necessary that $b_{ij}^{\left( 2 \right)} = - b_i^{\left( 1 \right)} - b_j^{\left( 1 \right)} < 0$. If a similar relation holds for pairs *ik* and *jk*, the effect of the triple mutant *ijk* can be canceled by choosing $b_{ijk}^{\left( 3 \right)} = b_i^{\left( 1 \right)} + b_j^{\left( 1 \right)} + b_k^{\left( 1 \right)} > 0$. In this way, an entire landscape can be constructed where all multiple mutants are ‘neutral’, and the Taylor coefficients alternate in sign and increase in magnitude with increasing order.

### Inferring the number of phenotypes underlying increased resistance and TEM protein levels

**Fig. 6:**
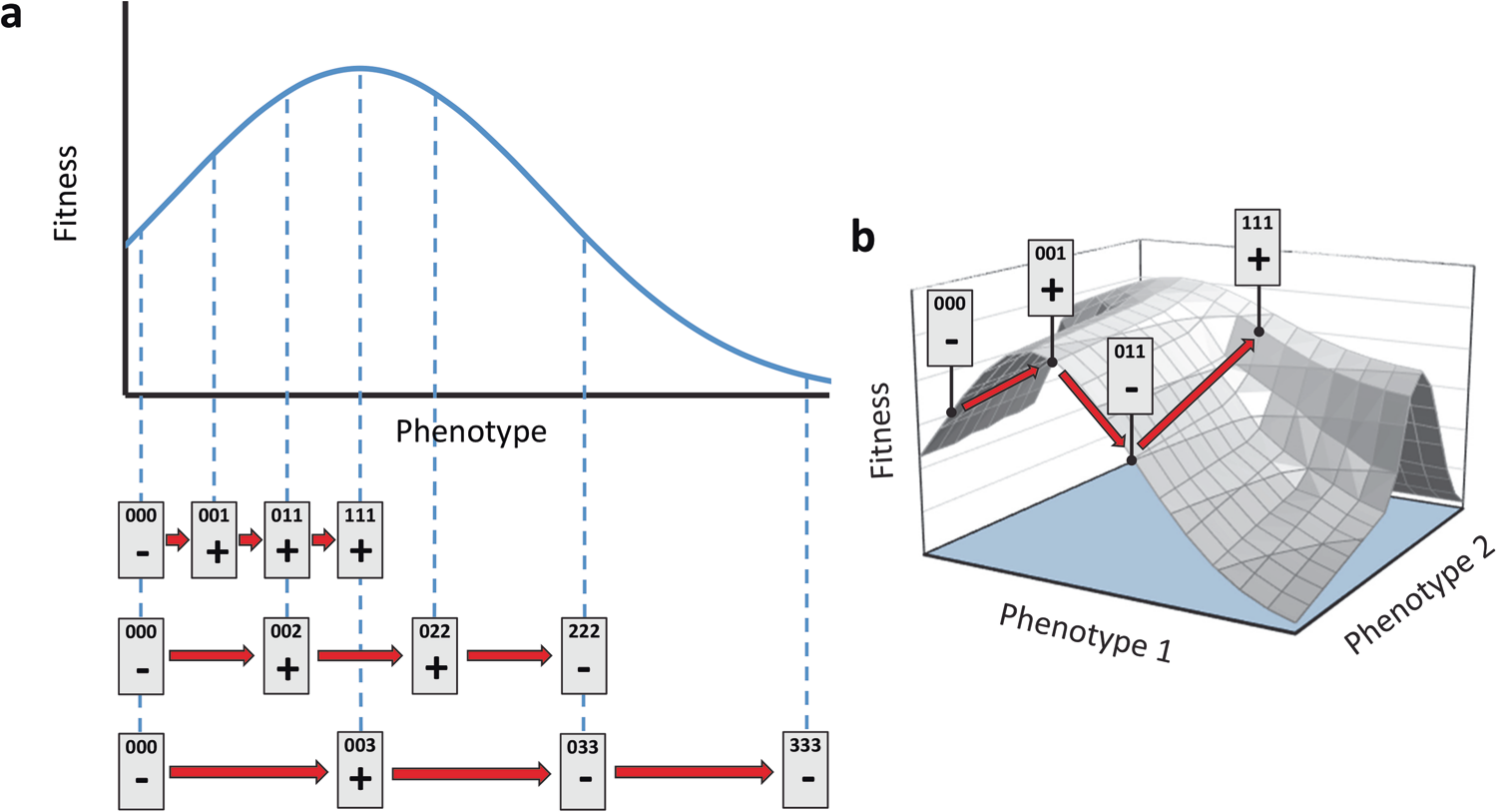
In **a**, we illustrate the nonlinear relationship between a one-dimensional phenotype with a single optimum and fitness, for beneficial mutations with different magnitudes with respect to the distance to the unique fitness optimum. When the effects of mutations on the phenotype are additive and the optimum is not overshot, addition of all mutations along a mutational trajectory (i.e., 000 → 001, etc.) always leads to higher fitness than the ancestor (000) as denoted by a plus sign. As the optimum is overshot, mutational trajectories lead to fitness values that are no longer higher than the ancestor. However, if there is a one-dimensional phenotype-fitness map with a single optimum, along a mutational trajectory all the mutations with a higher fitness than the ancestor must be aggregated. Mutational trajectories starting from the ancestor and adding beneficial mutations that cause decreases in fitness followed by increases in fitness require a multi-dimensional phenotype, as illustrated in **b**, or the unlikely case of a one-dimensional phenotype with multiple optima

All the mutations in the resistance landscape (R9*, A17*, G87*, and E89*) increase functional TEM protein levels, whilst not significantly affecting RNA levels. All these mutations therefore act post-transcriptionally, and within the total set of 20 mutations, their behavior appears to be comparable. The resistance landscape reveals strong epistatic interactions, but is it also informative about the dimensionality of the phenotypes affected by these four mutations? In the spirit of a recent approach that infers epistatic interactions from the rank ordering of fitness values in a landscape (Crona et al. 2017), we devised a simple test for the existence of a one-dimensional phenotype underlying the observed resistance effects. The basic idea is illustrated in Fig. 6. If there is a one-dimensional phenotype with additive mutational effects and a single resistance optimum, any pathway along which these beneficial mutations are added sequentially is either monotonically increasing in resistance or displays one maximum. Although strong interactions can result from overshooting the optimum of a one-dimensional phenotype-resistance map (Rokyta et al. 2011; Hwang et al. 2017), pathways along which the sign of resistance effect changes twice are ruled out. They require either a two-dimensional phenotype or an implausible one-dimensional phenotype-fitness map with several optima.

To implement this approach in our setting, it is sufficient to distinguish between alleles that have significantly higher resistance than TEM-1 and those that do not. The latter class comprises TEM-1 along with the two synthetically neutral alleles R9*/G87* and R9*/G87*/E89*. Since all single mutant alleles as well as the quadruple mutant R9*/A17*/G87*/E89* display elevated resistance, any pathway from TEM-1 to the quaduple mutant that contains at least one of the synthetic neutrals violates the constraint imposed by a one-dimensional phenotype. Out of the total number of 4! = 24 pathways, there are 4 that contain R9*/G87*, 6 that contain R9*/G87*/E89*, and 2 that contain both. Altogether 8 out of 24 pathways are therefore incompatible with a one-dimensional additive phenotype. Note that this result is concordant with measurements of total TEM protein levels for the landscape (Fig. 4c), where it is shown that the low-resistance R9*/G87* and R9*/G87*/E89* alleles do not have increased TEM protein levels and are not overshooting an optimum. We conclude that even for a subset of mutations that increase functional TEM protein levels, there is strong evidence for a multi-dimensional phenotype underlying the effects on protein level and resistance. Given that all these mutations appear to be acting post-transcriptionally, we find it surprising that such complex epistatic interactions arise and that multiple phenotypes are implicated.

## Discussion

The mechanisms by which synonymous mutations increase fitness are poorly understood, despite the mounting evidence that these mutations can meaningfully impact fitness. In our study, 5 out of 10 synonymous mutations significantly increased the levels of functional TEM-1 β-lactamase enzyme without altering TEM transcript levels. The TEM-1 allele has very low activity against CTX, but the synonymous mutations resulted in up to 7.5-fold increases in nitrocefin hydrolysis rates, reflecting similar increases in functional protein levels. It has previously been shown that both synonymous and non-synonymous mutations in the TEM coding region can affect TEM protein levels (Zalucki et al. 2008; Firnberg et al. 2014). We found a good correlation between TEM protein levels and nitrocefin hydrolysis rates, but no effect on TEM messenger, suggesting these synonymous mutations could act by increasing translation rates or by increasing TEM protein stability, e.g., by subtly affecting protein folding. In another study it was shown that effects on TEM protein levels were probably not due to differences in protein misfolding, as there was no relationship between total TEM protein and the fraction of TEM in the soluble and insoluble protein fractions (Firnberg et al. 2014). We therefore cautiously favor the hypothesis that at least some synonymous mutations affect translation rates.

We found that some non-synonymous mutations also affect TEM protein levels, a surprising result because we had anticipated that their effects would have been mediated by changes in protein structure affecting thermodynamic stability or catalytic efficiency. Some of these mutations confer similar increases in TEM (functional) protein levels and resistance to CTX as the synonymous mutations, suggesting that the primary mechanism by which these mutations act is by increasing TEM functional protein levels, rather than through changes in protein structure. By contrast, the F72V mutation appears to act by increasing TEM transcript levels, something we have not observed for the synonymous mutations. Our observations therefore underscore that there are different mechanisms that do not depend primarily on changes in protein structure by which mutations can affect an organism’s phenotype and fitness, and that the benefits of non-synonymous mutations depend partially on the same mechanism by which synonymous mutations act (Bartoszewski et al. 2010).

Synonymous mutations in TEM have been reported to affect not only resistance but also fitness in a study that measured TEM allele frequencies in bulk competitions on plates with antibiotics (Firnberg et al. 2014). We also have previously reported the finding that synonymous mutations (e.g., L40* and A184*) can increase the fitness and CTX resistance of the TEM-19 allele (containing the largest-effect single-nucleotide mutation, G238S; van Dijk et al. 2017). For the synonymous mutations in our present study, we found an inverse correlation between functional TEM protein levels and fitness in the absence of antibiotics, suggesting these mutations indeed have pleiotropic effects on competitive ability. A trade-off between TEM expression and competitive fitness may partly explain why expression-increasing synonymous mutations are not ubiquitously observed in experimental evolution or in clinical settings (Salverda 2008; Salverda et al. 2011). These fitness data also suggest an alternative explanation for the benefits of alleles with high TEM protein levels: classic microbiology studies have shown that reductions in growth may enhance resistance to beta-lactams (Hobby et al. 1942; Tuomanen et al. 1986), and mutations increasing TEM protein levels might increase resistance by slowing down growth. The two mechanisms—an increase in TEM protein and the concomitant reduction in growth rate—are probably compatible with each other, making it possible that they both play a role.

Surprisingly, some alleles without increased functional TEM levels—in particular L139*—had a higher fitness than TEM-1. For L139*, the mechanisms that lead to both higher CTX resistance and higher fitness in the absence of antibiotics are not clear. The higher growth rate of this allele could be unrelated to the increase in resistance (i.e., a synergistically pleiotropic effect) or even detrimental to resistance (Tuomanen et al. 1986). Conversely, the high fitness in the absence of CTX we have observed might be indirectly linked to increased resistance to CTX. We speculate that this mutation might allow cells to maintain unchanged functional TEM protein levels while lowering the metabolic costs of TEM expression, hereby freeing up cellular resources that can be redirected to growth or survival. For many of the synonymous mutations in our panel, the mechanisms leading to resistance remain unclear whilst they do not appear to be related to increased TEM expression.

We found very strong epistatic interactions between four of the synonymous mutations, which were stronger than for two resistance landscapes that we previously analyzed, in which non-synonymous mutations with either large or small effects on resistance to CTX were studied (Schenk et al. 2013). This observation may surprise, given that in general synonymous mutations will be more restricted in their possible phenotypic effects. In this specific case, all four mutations act post-transcriptionally to increase TEM expression, further restricting their mechanisms of action. Others have reported that attempts to engineer sequences with “optimal” or “non-optimal” codons can give highly unexpected results, and appear to be driven by context-dependent effects on protein folding, such as ribosome pausing (Agashe et al. 2012).

All third-order Taylor coefficients for the resistance landscape were positive whilst the second- and fourth-order coefficients were negative, a result possibly linked to the occurrence of synthetically neutral combinations, as the higher-order effects need to cancel out those of lower orders. A neutral pattern in which these effects balance each other out exactly seems improbable, so how can this result be explained? In this particular case, if the phenotype on which these mutations act—TEM protein expression—drops to levels below those of the wild-type TEM-1 allele, a major effect on resistance is unlikely; TEM-1 has a very low activity against CTX and its complete absence only leads to a minor decrease in resistance (Schenk et al. 2012). Empirical measurements of total TEM protein levels suggests the synthetically neutral combinations have lower expression levels than TEM-1, supporting the suggestion that these higher-order effects do not balance each other exactly at the level of the underlying phenotype.

Inspired by previous work (Rokyta et al. 2011; Crona et al. 2017), we used the empirical resistance landscape for four beneficial synonymous mutations to explore the dimensionality of their underlying phenotype. By assuming that the mutations acting on the phenotype combine additively, we were able to reject the idea of a one-dimensional phenotype that maps to resistance or functional protein level through a unimodal function. Therefore, our results suggest that these four synonymous mutations exert their beneficial effect via multiple post-transcriptional mechanisms leading to increased levels of functional TEM-1 protein. Some of the beneficial synonymous mutations we have studied appear to be mediated by effects other than increased functional TEM protein levels. Our results therefore emphasize the plurality of mechanisms by which synonymous mutations can affect antibiotic resistance.

Epistasis has been defined loosely as “the surprise at the phenotype when mutations are combined” (Weinreich et al. 2013). The element of surprise applies well to our findings here too. Not only have we been surprised to find beneficial effects of synonymous mutations but also by their strong epistatic interactions, stronger than observed for non-synonymous mutations of similar or larger benefit in the same gene. Some of the mutations appear to show their benefit by increasing the level of protein of what is in this context a lousy enzyme, for which different mechanisms seem to be involved, which may also explain the beneficial effect of non-synonymous mutations of similar effect size.

### Data archiving

All data have been deposited in Dryad (10.5061/dryad.hp370m2).

## Electronic supplementary material

File with supplementary text, figures and tables
