# Supplementary material for: Unraveling the causes of adaptive benefits of synonymous mutations in TEM-1 β-lactamase
Source: Heredity (Edinb). 2018 Jul 2;121(5):406–21. doi: 10.1038/s41437-018-0104-z (PMC6180035; doi:10.1038/s41437-018-0104-z)
Supplement: Supplementary file 1 — File with supplementary text, figures and tables [file 41437_2018_104_MOESM1_ESM.pdf]

SUPPLEMENTARY ONLINE INFORMATION

Appendix 1: No evidence stress responses are being affected by the panel of mutations

Indicator genes were *rpoS* for the general stress response, *spoT* and *relA* for the stringent response, and *sulA* for the SOS response. We also considered expression levels of *hipA*, to gauge activation levels of this toxin/anti-toxin system. Overall, there were no significant differences in expression levels of any of these genes for different TEM alleles, except *rpoS* (Table S4). In *post hoc* tests, none of the mutated TEM alleles differed significantly from TEM-1 in their levels of stress-response indicators (Figure S3). Expression levels of *spoT, relA, sulA* and *hipA* are similar, whereas *rpoS* expression is not.

We noted a significant correlation between *rpoS* and TEM messenger levels (Pearson correlation = 0.760, $N = 21$, $p < 0.001$), suggesting a possible role of stress induction. Overall the differences between the entire panel and the control plasmid without TEM (pACSE3) are marginal (Figure S3), suggesting that any effect of TEM expression on *rpoS* is subtle. Nevertheless, we generated a Δ*rpoS* strain and measured the resistance of two synonymous TEM alleles that showed relatively high levels of *rpoS* expression (E89* and G87*). Overall, resistance to CTX and other antibiotics was lower in the Δ*rpoS* background, whilst these two alleles still showed the same relative improvement compared to TEM-1 (Figure S4). We therefore discarded the idea that synonymous mutations induce stress responses and hereby increase resistance to CTX.

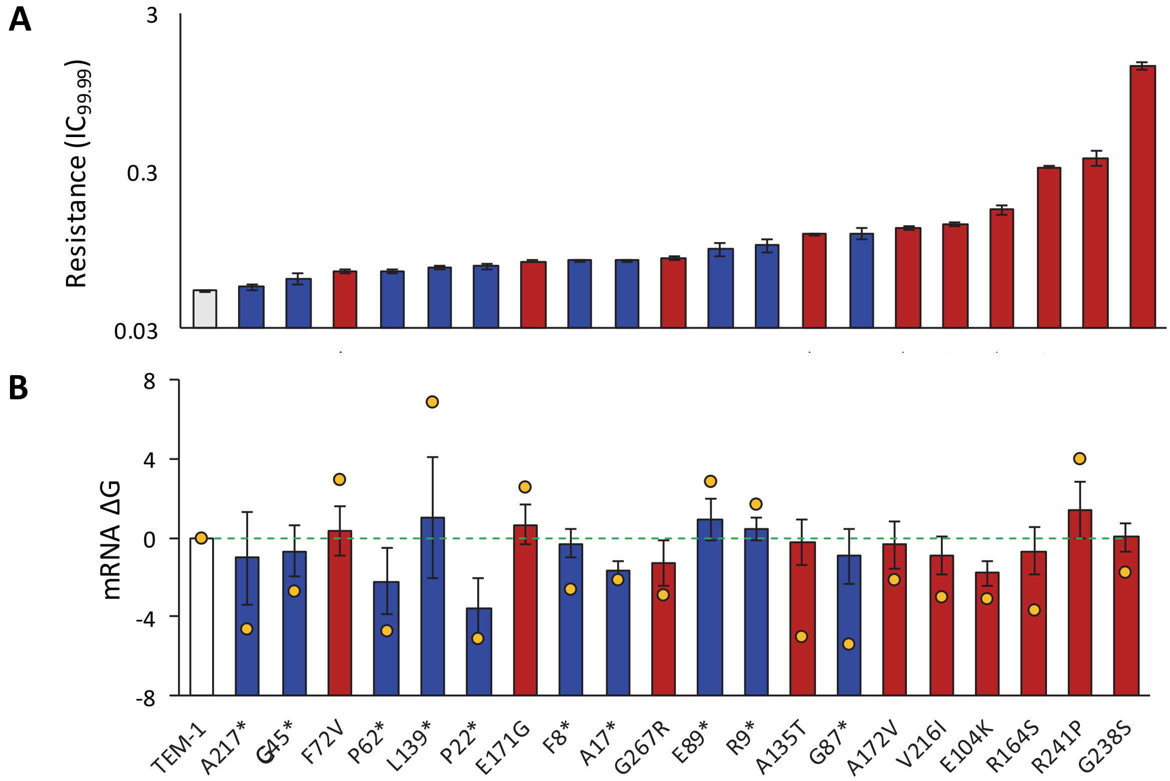

Figure S1: An overview of the mRNA ΔG values. For all panels, blue data correspond to the synonymous mutations and red data to the non-synonymous mutations. In panel A, resistance against CTX is given in µg/mL [$IC_{99.99}$] for the whole panel for a comparison. In panel B, the change in mRNA ΔG compared to TEM-1 is given, as determined by RNAfold on a sliding 45 bp window. Bars and error bars give the mean and standard error (N = 45) of the change in all windows overlapping with the mutation, whilst the yellow circles indicate the largest deviation from TEM-1.

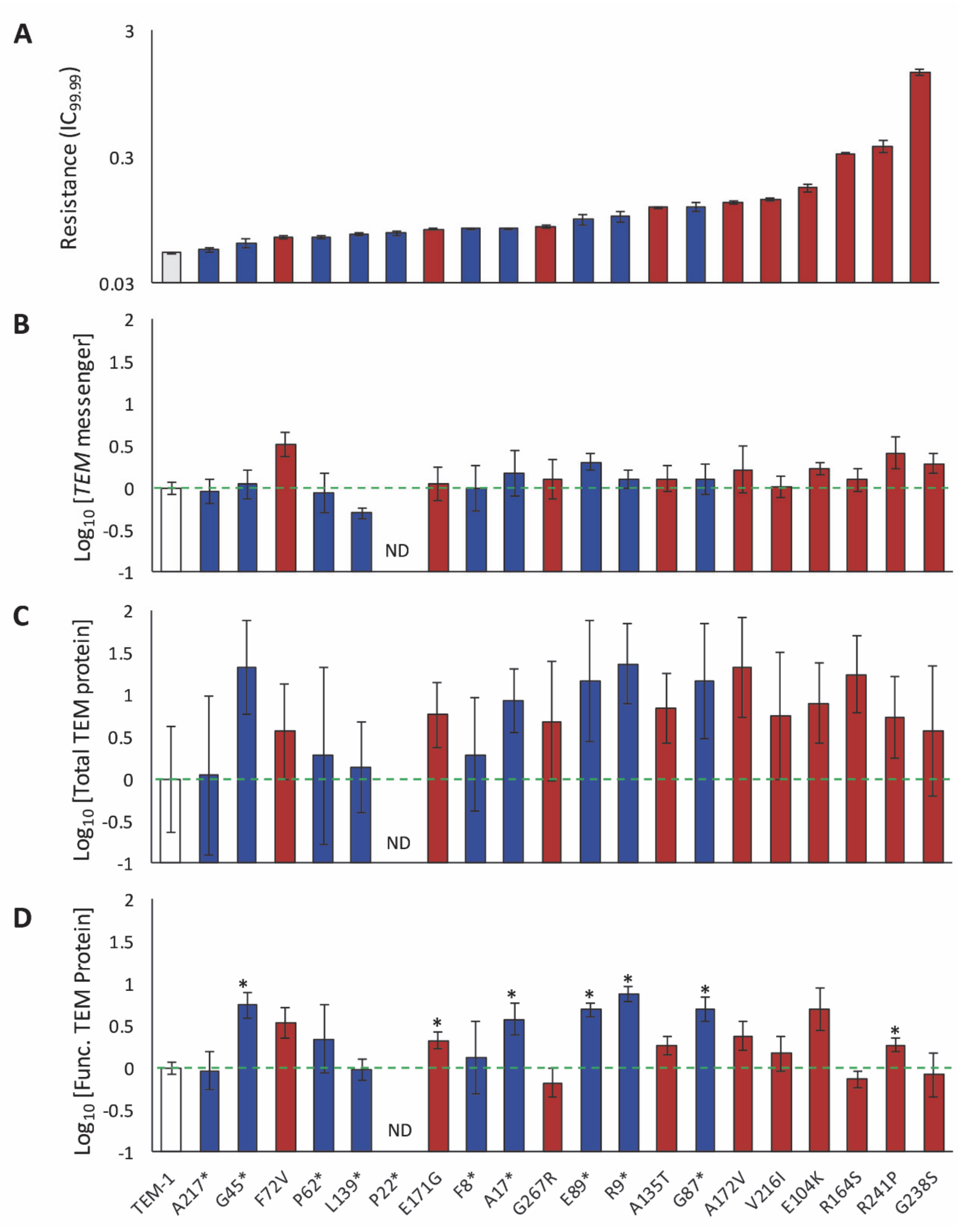

Figure S2. An overview of TEM expression and activity for the panel of synonymous and non-synonymous mutations, with values relative to TEM-1. For all panels, blue data correspond to the synonymous mutations and red data to the non-synonymous mutations, while error bars represent the standard error of the mean ($N = 3$). The dotted line indicates TEM-1 expression levels. In panel A, resistance against CTX is given in µg/mL [$IC_{99.99}$] for the whole panel. TEM messenger levels (Panel B), total TEM protein (Panel C) and functional TEM protein (Panel D) are given relative to TEM-1. For expression measures, we tested for pairwise differences between TEM-1 and all other alleles with an independent samples *t*-test employing Benjamini-Hochberg correction. Significant differences were found only for functional TEM protein levels and are marked with an asterisk. Overall, there are statistically significant differences for TEM messenger and functional protein levels (Table S4). Due to recurring deletions in the pACTEM plasmid, no data (ND) are available for P22*.

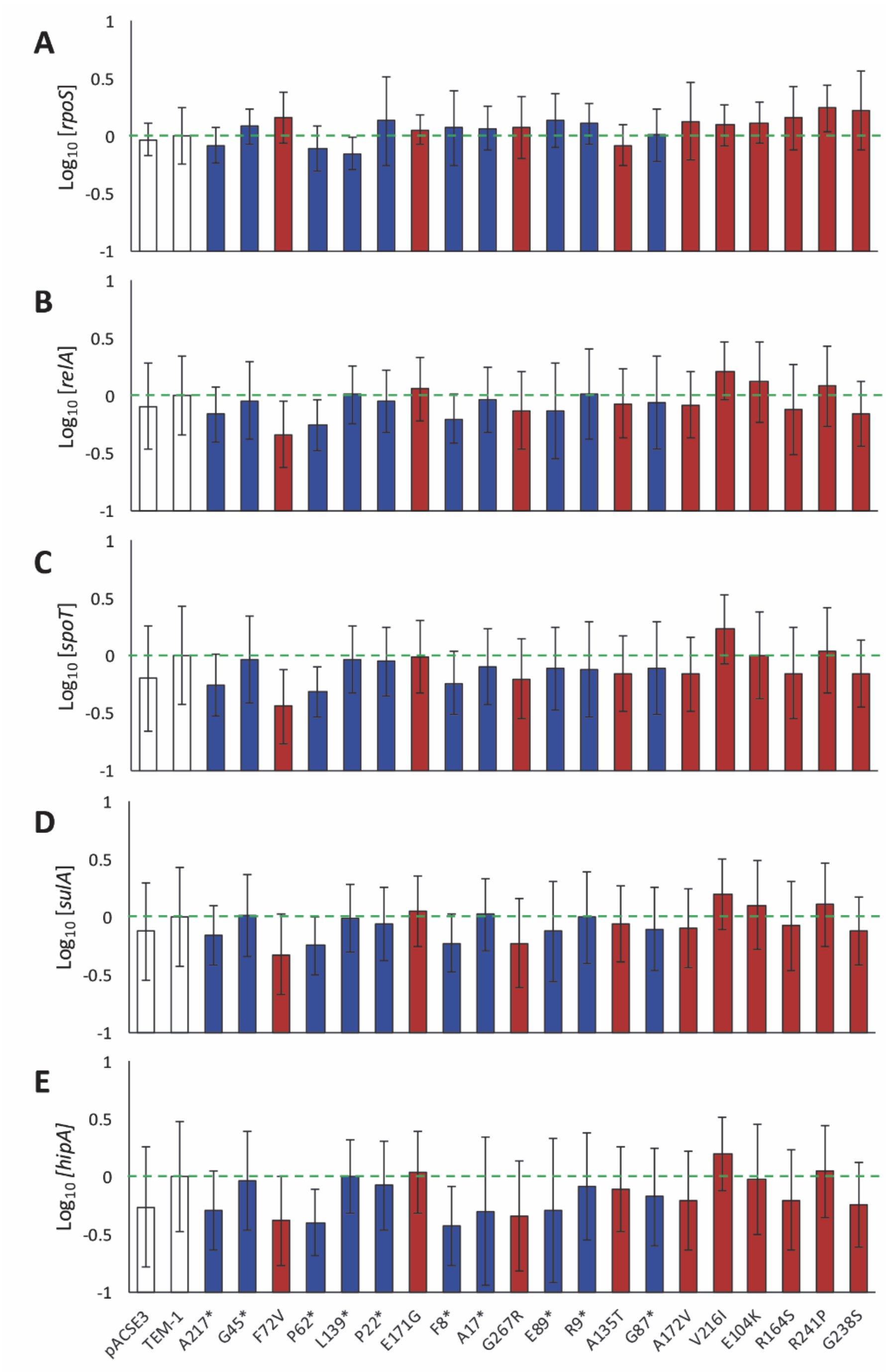

Figure S3: RT-qPCR measurements of expression levels for transcripts associated to *E. coli* stress responses. For all panels, the $\log_{10}$ gene expression, normalized to the TEM-1 control, is given, with error bars representing the standard error the mean ($N = 3$). White bars are control samples (pACSE3 control of plasmid with no TEM insert, and the wild-type TEM-1), blue bars are synonymous mutations and red bars are non-synonymous mutations. TEM alleles are ranked by fitness, with the lowest resistance variant being depicted on the left. Gene expression was measured for *rpoS* (general stress response, panel A), *relA* (stringent response, Panel B), *spoT* (stringent response, Panel C), *sulA* (SOS response, Panel D) and *hipA* (High persistor toxin/anti-toxin). Note how the signals of *relA, spoT, sulA* and *hipA* are highly correlated, whereas *rpoS* gives a different pattern. We tested for pairwise differences between TEM-1 and all other alleles with an independent samples *t* tests and a Benjamini-Hochberg correction, and found no significant differences.

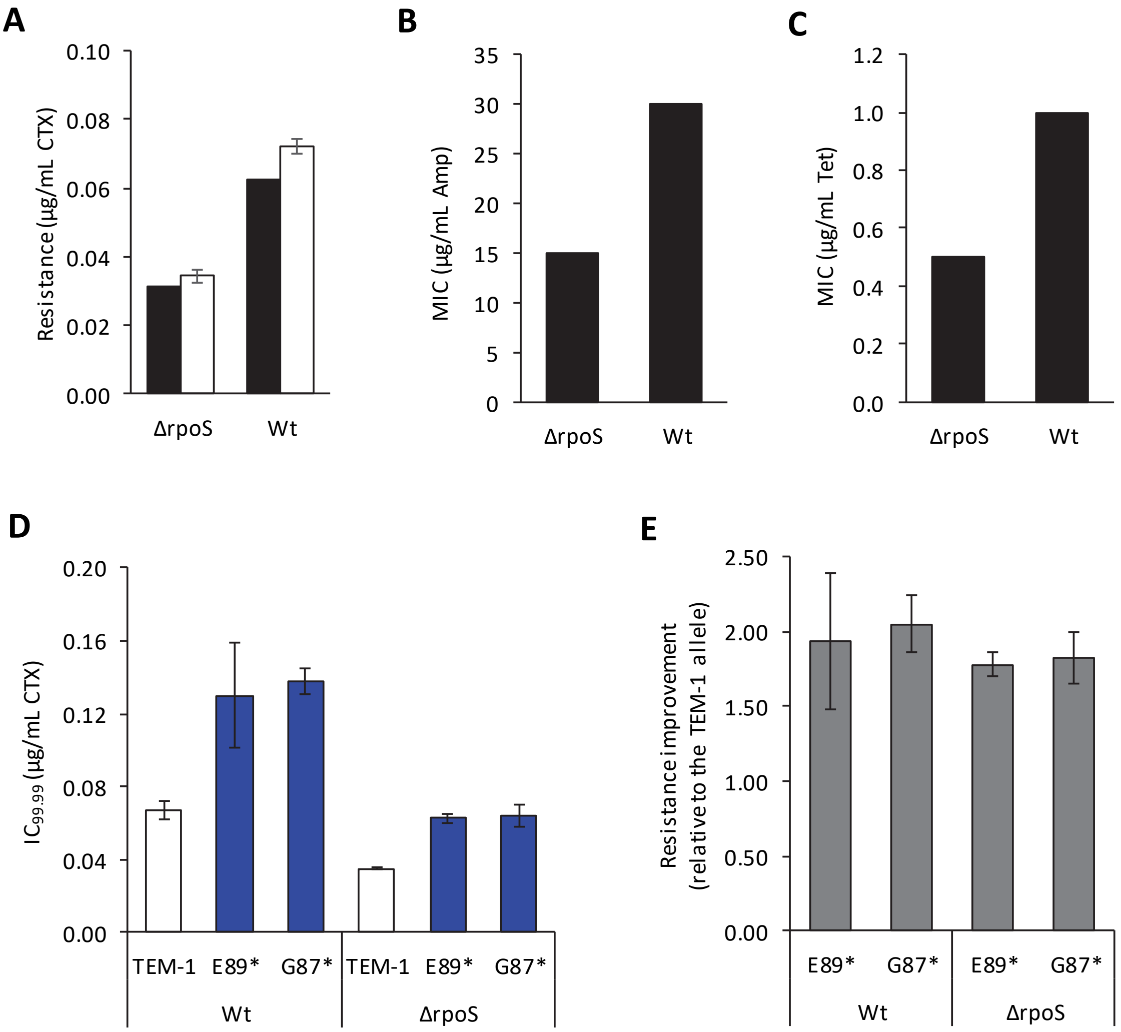

Figure S4: In panels A-C, the minimal inhibitory concentration (MIC) of wild-type and Δ*rpoS* *E. coli* for three antibiotics is given (black bars). For CTX, the $IC_{99.99}$ was also determined (white bars in panel A). In panel D, $IC_{99.99}$ values for wild-type and Δ*rpoS* cells carrying different TEM alleles are given. In panel E, the relative increase in resistance for the two synonymous mutations in both backgrounds is shown. In panels A, D and E error bars represent the standard error of the mean and $N = 2$, whereas the number of replicates for MIC assays is 3-5. Despite the overall decrease in resistance in the Δ*rpoS* background, the two synonymous alleles confer similar increases in resistance in both backgrounds, suggesting the beneficial effects of these mutations are independent of *rpoS*.

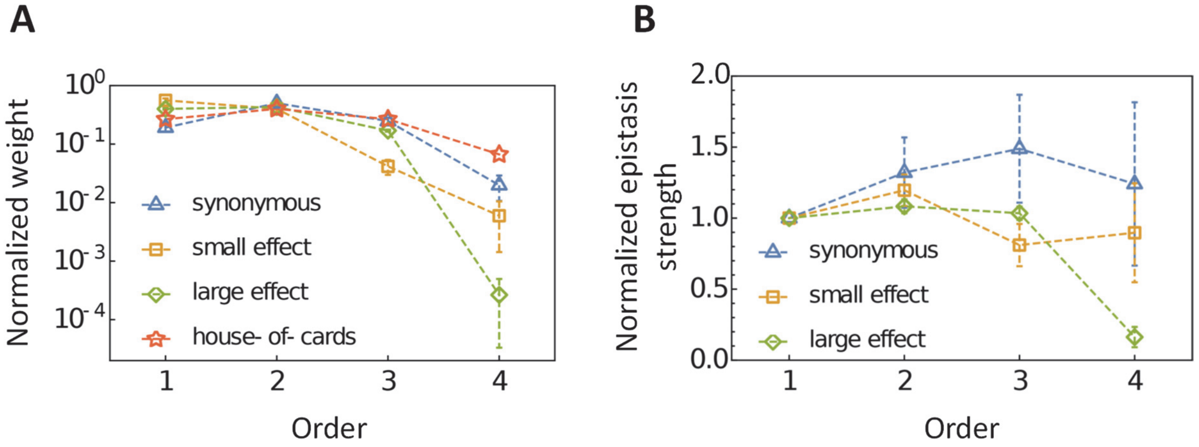

Figure S5: In Panel A, Fourier spectra of the synonymous resistance landscape and the two non-synonymous landscapes (Schenk *et al.*, 2013), with error bars denoting the standard error of the mean ($N = 3$). The prediction for a maximally rugged House-of-cards landscape is also depicted. In Panel B, normalized Taylor coefficients of the synonymous and the two non-synonymous landscapes are given.

Table S1: qPCR primers

Gene	Primer	Sequence	Notes
TEM	Forward	5’-AAGCCATACCAAACGACGAG	
	Reverse	5’-TGCCGGGAAGCTAGAGTAAG	
cysG	Forward	5’-TTGTCGGCGGTGGTGATGTC	[1]
	Reverse	5’-ATGCGGTGAACTGTGGAATAAACG	
rrsA	Forward	5’-CTCTTGCCATCGGATGTGCCCA	[2]
	Reverse	5’-CAGTGTGGCTGGTCATCCTCTCA	
TcR	Forward	5’-GTCCAGGCAGGTAGATGACG	
	Reverse	5’-CGGTCCAGTGATCGAAGTTAG	
rpoS	Forward	5’-CAGAGCATCGTCAAATGGCTG	
	Reverse	5’-TTCCAGTGTTGCCGCTTCG	
spoT	Forward	5’-CCGTAAGCTGAATGAAATCCC	
	Reverse	5’- CATTGCGTTACCAAGTCCGA	
relA	Forward	5’- CGGGACATGAAGACCGGATT	[3]
	Reverse	5’- ATCTCTTCCTGCCACGCAAT	
sulA	Forward	5’-ACATCGTTCTTCGTCGTTCTC	
	Reverse	5’-ATAGACAACTTCACTGATAAGCCC	
hipA	Forward	5’-GACTTTGCCAGAATGATTCCAG	
	Reverse	5’-TTAACCGTCCATGCAACCTC	

[1] Zhou K, Zhou L, Lim Q ’En, Zou R, Stephanopoulos G, Too H-P (2011). Novel reference genes for quantifying transcriptional responses of Escherichia coli to protein overexpression by quantitative PCR. *BMC Mol Biol* **12**: 18.

[2] Peng S, Stephan R, Hummerjohann J, Tasara T (2014). Evaluation of three reference genes of Escherichia coli for mRNA expression level normalization in view of salt and organic acid stress exposure in food. *FEMS Microbiol Lett* **355**: 78–82.

[3] Brown DR, Barton G, Pan Z, Buck M, Wigneshweraraj S (2014). Nitrogen stress response and stringent response are coupled in Escherichia coli. *Nat Commun* **5**: 1–8.

Table S2: The panel of TEM alleles selected fort this study[a]

Mutation ID #	Mutation	Original codon	Modified codon	Replacement	Clinical isolates[b]	Experimental evolution	Resistance level IC99.99 (µg Ctx/mL) ± S.D.	Improvement in resistance to CTX
1	*g706a*	ggt	agt	G238S	34	Yes	1.41 ± 0.15	27
2	*g713c*	cgt	cct	R241P	0	No	0.363 ± 0.067	7.0
3	*c484a*	cgt	agt	R164S	30	Yes	0.320 ± 0.008	6.2
4	*g304a*	gag	aag	E104K	46	Yes	0.171 ± 0.019	3.3
5	*g640a*	gtt	att	V216I	0	Yes	0.138 ± 0.005	2.7
6	*c509t*	gcc	gtc	A172V	0	No	0.131 ± 0.004	2.5
7	*g255a*	ggg	gga	G87*	0	-[c]	0.121 ± 0.016	2.3
8	*g397a*	gcc	acc	A135T	0	No	0.119 ± 0.005	2.3
9	*t21a*	cgt	cga	R9*	1	-[c]	0.101 ± 0.016	2.0
10	*g261a*	gag	gaa	E89*	2	-[c]	0.096 ± 0.016	1.9
11	*g787c*	ggg	cgg	G267R	0	Yes	0.085 ± 0.004	1.6
12	*c18t*	ttc	ttt	F8*	42	-[c]	0.081 ± 0.003	1.6
13	*g45t*	gcg	gct	A17*	0	-[c]	0.081 ± 0.002	1.6
14	*a506g*	gaa	gga	E171G	0	No	0.080 ± 0.002	1.5
15	*t60c*	cct	ccc	P22*	0	-[c]	0.075 ± 0.006	1.4
16	*g411a*	ctg	cta	L139*	0	-[c]	0.074 ± 0.004	1.4
17	*c180t*	ccc	cct	P62*	0	-[c]	0.069 ± 0.004	1.3
18	*t208g*	ttt	gtt	F72V	0	No	0.069 ± 0.003	1.3
19	*t129c*	ggt	ggc	G45*	0	-[c]	0.062 ± 0.008	1.2
20	*a645t*	gca	gct	A217*	0	-[c]	0.055 ± 0.003	1.1
	none			pACTEM1	-	-	0.052 ± 0.002	1.0
	none			pACSE3	-	-	0.033 ± 0.003	0.6

[a] Table adapted from Schenk et al. 2012. [b] Number of times present in 179 clinical samples. [c] Record is incomplete for synonymous mutations.

Table S3: Predicted codon influence on expression.

Mutation ID #	Mutation	Replacement	Improvement in resistance to CTX	Codon influence[a] for original codon ±SEM	Codon influence[a] for modified codon ±SEM	t_{120}[b]	*P* value[c]	Increase or decrease codon influence
1	*g706a*	G238S	27	16.068 ±2.656	9.656 ±3.549	1.448	0.150	
2	*g713c*	R241P	7.0	13.025 ±3.349	5.702 ±3.870	1.431	0.151	
3	*c484a*	R164S	6.2	13.025 ±3.349	9.656 ±3.549	0.690	0.491	
4	*g304a*	E104K	3.3	-4.618 ±1.742	2.211 ±1.975	2.593	0.011*	>
5	*g640a*	V216I	2.7	14.475 ±2.387	11.477 ±1.763	1.010	0.314	
6	*c509t*	A172V	2.5	-9.018 ±1.190	-9.390 ±2.158	0.151	0.372	
7	*g255a*	G87*	2.3	-17.123 ±3.044	4.077 ±2.511	5.372	< 0.001*	>
8	*g397a*	A135T	2.3	-9.018 ±1.190	-3.316 ±2.189	2.289	0.024	
9	*t21a*	R9*	2.0	13.025 ±3.349	1.813 ±5.502	1.741	0.084	
10	*g261a*	E89*	1.9	-4.618 ±1.742	20.022 ±1.597	10.426	< 0.001*	>
11	*g787c*	G267R	1.6	-17.123 ±3.044	-24.521 ±2.668	1.828	0.070	
12	*c18t*	F8*	1.6	-9.441 ±2.480	8.187 ±1.918	5.623	< 0.001*	>
13	*g45t*	A17*	1.6	-8.825 ±1.560	11.955 ±2.531	6.989	< 0.001*	>
14	*a506g*	E171G	1.5	20.022 ±1.597	4.077 ±2.512	5.423	< 0.001*	<
15	*t60c*	P22*	1.4	5.702 ±3.870	-16.746 ±2.730	4.740	< 0.001*	<
16	*g411a*	L139*	1.4	-8.391±1.221	-2.318 ±4.360	1.341	0.182	
17	*c180t*	P62*	1.3	-16.746 ±2.730	5.702 ±3.870	4.740	< 0.001*	>
18	*t208g*	F72V	1.3	8.187 ±1.918	14.475 ±2.387	2.053	0.042	
19	*t129c*	G45*	1.2	16.068 ±2.646	-7.880 ±1.396	8.006	< 0.001*	<
20	*a645t*	A217*	1.1	10.844 ±2.500	11.955 ±2.531	0.324	0.747	

[a] Codon influence on expression according to the single-parameter binary logistic regression index, see Boël et al. (2016): Figure 3 and supplementary materials. [b] Number of observations and d.f. based on the average number of data points per codon. [c] Significant *P* values after Benjamini-Hochberg correction for multiple comparisons are marked with an asterisk.

Table S4: Welch test results for expression measures for the panel.

Type	Measurement	Test statistic	df1, df2	*P* value
Stress response	*rpoS*	3.802	20, 14.475	0.006
	relA	1.225	20, 15.538	0.347
	spot	2.218	20, 14.852	0.061
	sulA	1.317	20, 15.323	0.294
	hipA	1.797	20, 15.338	0.123
TEM	messenger	7.597	19, 14.623	< 0.001
	total protein	1.466	19, 14.653	0.231
	functional protein	11.009	19, 14.667	< 0.001

Stress response analyses were done on all data (except P22* allele), including the pACSE3 allele. As we expect no TEM signal for pACSE3, this variant was not included for analyses on TEM to avoid an arbitrary significant result.

Table S5: Fourier and Taylor coefficients for the synonymous resistance landscape.

Mutation in TEM				Coefficient	
R9*	A17*	G87*	E89*	Fourier	Taylor
0	0	0	1	−1.05754	0.92924
0	0	1	0	0.57953	1.47635
0	1	0	0	−3.1817	0.645316
1	0	0	0	−0.820921	0.871769
0	0	1	1	−2.14433	−1.58719
0	1	0	1	−0.131142	−0.78624
0	1	1	0	1.12728	−1.27697
1	0	0	1	0.176057	−0.578779
1	0	1	0	−3.87497	−2.39687
1	1	0	0	3.33074	−0.297794
0	1	1	1	−1.78579	1.47064
1	0	1	1	−1.26314	1.20932
1	1	0	1	−0.0725435	0.614014
1	1	1	0	−3.29388	2.22468
1	1	1	1	−1.15549	−1.15549
